# Feasibility of supervised self-testing using an oral fluid-based HIV rapid testing method: a cross-sectional, mixed method study among pregnant women in rural India

**DOI:** 10.7448/IAS.19.1.20993

**Published:** 2016-09-12

**Authors:** Archana Sarkar, Gitau Mburu, Poonam Varma Shivkumar, Pankhuri Sharma, Fiona Campbell, Jagannath Behera, Ritu Dargan, Surendra Kumar Mishra, Sunil Mehra

**Affiliations:** 1 Research, Innovations, and Monitoring Unit, MAMTA Health Institute for Mother & Child, New Delhi, India; 2 Program Impact Unit, International HIV/AIDS Alliance, East Sussex, UK; 3 Division of Health Research, School of Health and Medicine, Lancaster University, Lancaster, UK; 4 Department of Obstetrics & Gynaecology, Mahatma Gandhi Institute of Medical Sciences, Wardha, India; 5 Faculty of Public Health and Policy, London School of Hygiene & Tropical Medicine, London, UK

**Keywords:** supervised HIV self-testing, pregnant women, India, acceptability, feasibility

## Abstract

**Introduction:**

HIV self-testing can increase coverage of essential HIV services. This study aimed to establish the acceptability, concordance and feasibility of supervised HIV self-testing among pregnant women in rural India.

**Methods:**

A cross-sectional, mixed methods study was conducted among 202 consenting pregnant women in a rural Indian hospital between August 2014 and January 2015. Participants were provided with instructions on how to self-test using OraQuick^®^ HIV antibody test, and subsequently asked to self-test under supervision of a community health worker. Test results were confirmed at a government-run integrated counselling and testing centre. A questionnaire was used to obtain information on patient demographics and the ease, acceptability and difficulties of self-testing. In-depth interviews were conducted with a sub-sample of 35 participants to understand their experiences.

**Results:**

In total, 202 participants performed the non-invasive, oral fluid-based, rapid test under supervision for HIV screening. Acceptance rate was 100%. Motivators for self-testing included: ease of testing (43.4%), quick results (27.3%) and non-invasive procedure (23.2%). Sensitivity and specificity were 100% for 201 tests, and one test was invalid. Concordance of test result interpretation between community health workers and participants was 98.5% with a Cohen's Kappa (k) value of *k*=0.566 with *p<*0.001 for inter-rater agreement. Although 92.6% participants reported that the instructions for the test were easy to understand, 18.7% required the assistance of a supervisor to self-test. Major themes that emerged from the qualitative interviews indicated the importance of the following factors in influencing acceptability of self-testing: clarity and accessibility of test instructions; time-efficiency and convenience of testing; non-invasiveness of the test; and fear of incorrect results. Overall, 96.5% of the participants recommended that the OraQuick^®^ test kits should become publicly available.

**Conclusions:**

Self-testing for HIV status using an oral fluid-based rapid test under the supervision of a community health worker was acceptable and feasible among pregnant women in rural India. Participants were supportive of making self-testing publicly available. Policy guidelines and implementation research are required to advance HIV self-testing for larger populations at scale.

## Introduction

HIV testing is a critical entry point for early identification and initiation of HIV treatment [1]. In addition, awareness of HIV status is an important factor in HIV prevention, including prevention of mother-to-child transmission [2]. However, most recent figures indicate that only 44% of pregnant women in low- and middle-income countries are tested for HIV; in India, this number is only 37% [3]. As a result, approximately 240,000 children in low- and middle-income countries are newly infected with HIV annually, most of them through mother-to-child transmission. This number is six times higher than the global target of less than 40,000 annual infections needed to virtually eliminate mother-to-child transmission of HIV [3,4].

Access to HIV testing by pregnant women is hindered by individual, social and structural factors. In India, these include low awareness of HIV testing services, poor understanding of ways to prevent mother-to-child transmission of HIV, poor perception of HIV risk, social and cultural barriers (such as low partner support), and fear of stigma and discrimination following disclosure [5,6]. Access to HIV testing and retention in care is further obstructed by factors related to the Indian health system, such as a lack of trained healthcare workers for antenatal HIV counselling [6], long distances to HIV testing facilities, especially in rural areas [7,8], and inequalities in antenatal care coverage and attendance [9].

To overcome some of these barriers, a range of technologies and operational approaches are required to increase uptake of HIV testing. One potential approach is HIV self-testing using a rapid diagnostic test. Tests can be blood-based, using samples from finger-stick tests, or saliva-based, using oral fluid for HIV testing. Most rapid HIV diagnostic tests can provide results in less than 30 minutes [10,11]. One example approved by the U.S. Food and Drug Administration is OraQuick^®^ (OraSure Technologies, Inc.), which can detect HIV in both blood and oral fluid samples.

Over the last 15 years, studies have examined supervised and unsupervised self-testing approaches in a range of settings (i.e. hospital and community) and population groups (i.e. the general population, health professionals and high-risk groups, including men who have sex with men) in both high- and low middle-income settings, such as USA, Canada, Spain, Singapore, Kenya, Malawi and India [12–16]. In previous studies, acceptability of oral fluid-based self-testing has been high, ranging from 74 to 95% in one systematic review [14], and sensitivity and specificity have been reported as 98.03 and 99.74%, respectively, for pooled results [17], although sensitivity was lower at 93.6%, in a recent large community study [13]. Available evidence suggests that acceptability of oral fluid-based self-testing is higher compared to blood-based testing. In a recent study in rural India, an oral fluid-based HIV test was preferred by 87% of participants for first-time testing and 60% of participants for repeat testing [18]. In addition, for HIV self-testing, a preference for the saliva-based test has been noted over the blood-based test in the USA and Australia [19] because it is non-invasive and pain-free [20].

Although several studies have examined the provision of rapid HIV testing to pregnant women [21], they have employed provider-initiated approaches in the Indian context [22]. Pant Pai *et al*. [23] examined provider-initiated oral fluid-based HIV testing during active labour and demonstrated high acceptance levels of 98%. However, self-testing was not explored in the study. Self-testing, particularly when it is non-invasive and oral fluid based, may provide an option for early HIV screening of pregnant women, especially during antenatal visits. This has important implications in India, where an estimated 29 million women give birth each year, and 14,000 HIV-infected babies are born to an estimated 38,000 HIV-positive pregnant women annually. This accounts for nearly 5% of overall HIV transmission nationally [24].

This study aimed to explore the acceptability, concordance and feasibility of supervised HIV self-testing among pregnant women attending an antenatal clinic in the outpatient department of a rural hospital, using a non-invasive rapid oral fluid-based HIV test. Specifically, it explored the feasibility of self-testing supervised by community health workers for hospital outreach in rural India, known as auxiliary nurse midwives, rather than staff nurses and doctors.

## Methods

### Study design

This was a cross-sectional mixed methods study exploring supervised self-testing through a semi-structured questionnaire, followed by in-depth interviews. The study was conducted between August 2014 and January 2015 and follows STROBE [25] and COREQ [26] guidelines for reporting quantitative and qualitative data, respectively.

### Study setting

The study was conducted at Kasturba Hospital in the Mahatma Gandhi Institute of Medical Sciences, Wardha, India. This is a tertiary-level government hospital located in the state of Maharashtra in western India, with a catchment area of nearly 100,000 people. The hospital caters mainly to people of low socio-economic status in the adjoining rural areas, whose main occupations are related to agriculture, small business and marginal labour. At this hospital, pregnant women who attend antenatal care are routinely offered HIV testing, with acceptance rates of nearly 90%. The hospital also acts as a referral centre for nearby towns in the states of Maharashtra and Andhra Pradesh, where HIV prevalence is 0.62 and 0.97%, respectively, and the literacy level among women is 75.4 and 59.7%, respectively [27,28].

### Study population and participant recruitment

This study targeted pregnant women over 18 years of age, in their first trimester of pregnancy, and were due to attend antenatal care at the hospital. Pregnant women were excluded from participation if they were under 18 years of age, had missed their antenatal care appointments, had oral ulcers, bleeding gums or other periodontal disease, abnormal vital signs (such as fever >38.5°C), or any other pregnancy complications that might have hindered informed consent.

### Data collection team

The study involved a data collection team of three health workers, who administered the semi-structured questionnaire and supervised the self-testing procedure at the hospital, and an additional three interviewers, who conducted follow-on in-depth interviews. The three health workers were auxiliary nurse midwives, who were part of the hospital outreach staff. In the Indian healthcare system, the auxiliary nurse midwife is the primary grassroots-level functionary, who is in direct contact with the community and provides preventive maternal and child healthcare services. As the frontline (female) health worker, the auxiliary nurse midwife is the central focus of all reproductive and child health programmes and is trained for 18 months to perform this role [29]. The three interviewers (PS, RD, PB), all females, were experienced researchers trained in research methods, medicine and social work, respectively, and their primary occupations at the time of the study were researcher, general practitioner and study coordinator, respectively. In addition, all authors of this study are trained and experienced researchers.

### Study procedures

The auxiliary nurse midwives and interviewers were oriented on the study objectives and protocol by three authors (AS, PS, PVS), and the auxiliary nurse midwives were trained on how to complete the questionnaire based on their own observations and participants’ responses to the questions, while maintaining confidentiality during interviews. Instruction guides available with each OraQuick^®^ test kit were used to orient the three auxiliary nurse midwives on the procedures for setting up the kit, collecting saliva samples, testing and interpreting results, and taking precautions for infection control and contamination. A pilot exercise was conducted during which the health workers practiced supervision of self-testing among a set of women, who were not part of the study sample. This was done to familiarize the auxiliary nurse midwives with the procedure, and to assess their skills, competency and ability to follow a standardized procedure for giving instructions, documenting challenges and interpreting results. Quality checks were periodically conducted (by PVS and RD) during the data collection.

During antenatal visits to the hospital, eligible women were approached directly, informed about the goals of the study, and invited to participate by the auxiliary nurse midwives. Those who expressed interest were required to provide consent. Each consenting participant was given comprehensive pre-test counselling on HIV, including: sexual and mother-to-child transmission of HIV; benefits of knowing their status; disclosure, discordance and risk of infection in sero-discordant relationships; HIV prevention; antiretroviral therapy (ART); confidentiality of testing; the right to refuse a test; and the importance of antenatal visits and institutional delivery. The auxiliary nurse midwives also explained to participants that HIV testing can be conducted using either a blood or saliva sample. Participants were then introduced to the procedure for oral fluid-based rapid HIV self-testing using the OraQuick^®^ kit and how to interpret the results. To orient participants on self-testing, a simplified version of the self-testing protocol with pictorial representation was used (attached as Supplementary file 1).

Following pre-test counselling, participants were asked to perform self-testing in a private room observed by the auxiliary nurse midwives. While participants waited for results, information on demographics, knowledge on HIV testing and acceptability of HIV self-testing were obtained through a semi-structured questionnaire administered verbally by the health workers in Hindi, Marathi, or Telugu, depending on participants’ preferred language.

Test results were observed and interpreted first by participants in a private room and then by the health worker independently. To determine the ability of women to interpret the test results accurately, participants were provided with three pictorial model test results (positive, negative and an invalid result). Specific instructions were given to auxiliary nurse midwives not to influence participants’ interpretation of results, in order to ensure that women interpreted their own results without any prompting by a health worker. The project coordinators (JB and PB) in the hospital were intermittently monitoring both auxiliary nurse midwives and pregnant women to ensure that the protocol was observed. Participants were instructed to alert the auxiliary nurse midwives once they had read and interpreted the results. Subsequently, the auxiliary nurse midwives recorded both their own interpretation of the results, as well as participants’. To assess the concordance of self-testing, inter-rater agreement was measured between the results of self-testing as interpreted by participants and as interpreted by auxiliary nurse midwives.

Post-test counselling related to the test result and future linkage to care was given to all participants by the auxiliary nurse midwives irrespective of their test results. In all cases, it was emphasized to participants that the results needed to be confirmed. Subsequently, participants were referred to the integrated counselling and testing centre (ICTC) for confirmatory HIV testing. Confirmation at ICTC was conducted using the standard national algorithm of three rapid HIV test kits [30]. All participants were linked to ICTC database using unique IDs to ensure that results were matched. Following the self-testing procedure, each participant was asked if they would be willing to provide further information on their experience and to indicate whether they would agree to be visited at home for an in-depth interview within five days of self-testing. As a result, a sub-sample of women was selected using convenience sampling for interviewing on the basis of their availability and willingness to participate. This sample was selected prospectively, and recruitment continued until data saturation was achieved.

### Data collection

A pre-tested, semi-structured questionnaire was used by the three auxiliary nurse midwives to obtain information from each participant on their demographic profile; knowledge of HIV testing; experiences of pre- and post-test counselling; and ease, acceptability and difficulties with self-testing. Most measures in the questionnaire were pre-determined based on existing literature, while allowing for user-defined measures. For example, to determine the reason for accepting the test, four main options were offered based on literature: (1) ease of performing the test, (2) perceived need for testing, (3) participants trust of the result and (4) other, which were user defined. An observation schedule was developed for the health workers to document the complete procedure for self-testing as carried out by participants, including the errors and inconsistencies.

In-depth interviews were conducted with pregnant women to obtain information on their experience of oral fluid-based rapid HIV self-testing. Interview guides aimed to situate participants’ experience of self-testing within a broader social context, including their decision to test, testing preference and future utilization of self-testing. Interviews were conducted at participants’ homes, at a time of their choice and in their local language (Hindi, Marathi or Telugu). Researchers had limited personal knowledge of, or established relationships with, participants, and vice versa. However, rapport was built between participants and interviewers prior to the interviews.

The interviewers kept field notes and safeguarded the privacy of the interviews by ensuring that non-participants were not present during the interviews. Interviewers probed ambiguous responses and conducted informal member checks verbally throughout the fieldwork as part of narrative accuracy checks. No repeat interviews were conducted. Interviews were audio recorded and lasted for an average of 30 minutes.

### Operational definitions

Acceptability was defined as the proportion of uptake of the oral fluid-based HIV test, where the numerator was the number of participants who chose to self-test, and the denominator was the number who were offered and consented to testing, computed as a percentage. Structured questions were also used to substantiate and assess acceptability. Sensitivity and specificity analysis of OraQuick^®^ HIV kit results with traditional ICTC HIV results were conducted. The index test was a self-test result as interpreted by a health worker. Reference standard tests were the confirmatory tests done for HIV at the ICTC. Concordance for self-testing was reported as the measure of agreement of the test result interpretation between a participant and a health worker, quantified as a percentage agreement and with the Cohen's Kappa (κ) inter-rater agreement.

Feasibility was assessed using criteria suggested by Pant Pai *et al*. [14], that is, the “documented completion of self-testing and counselling processes.” For assessment of feasibility in this study, observation of the test procedure followed by participants was captured through 13 steps, starting from opening the kits and concluding with interpretation of the results. For analysis, these steps were then merged into three components: (1) preparing the test kit, (2) taking the sample and doing the test and (3) reading and interpreting the result.

### Sample size

The number of participants predicted to accept the test was at least 74%, based on the literature on self-testing reporting a minimum of 74% acceptability for oral testing in different settings and populations [14]. To estimate the proportion in this study at 95% confidence level with 10% margin of error, the minimum sample size of 135 pregnant women was required. An allowance was made for non-response and unusable data, as has been employed elsewhere [31], which generated an overall sample size target of at least 182 was derived, which approximates that used in other feasibility studies of oral HIV self-testing [32].

### Analysis

For the quantitative survey, descriptive statistics were computed related to participants’ knowledge, attitudes and experiences regarding self-testing. To estimate the concordance between participants’ and auxiliary nurse midwives’ readings, inter-rater agreement measured using Cohen's Kappa (κ). In this calculation, invalid results were included, as in other studies [33,34]. For analysis of self-testing sensitivity and specificity, pairs with invalid test results were excluded. Test kit sensitivity and specificity were computed from test results identified by auxiliary nurse midwives compared to confirmatory test results. All statistical analyses were performed using IBM SPSS Statistics v.22.

Qualitative data were first translated into English, and transcripts were analyzed through an inductive approach in which themes were identified during the course of analysis [35]. Responses were coded manually by two authors (AS and PS) and similar responses grouped together. Coding concepts were grouped into different categories and subsequently linked and compared through inductive analysis [36]. An initial list of thematic codes was generated from interviews, then refined and clustered, based on similarities [36]. Codes were then systematically classified and organized under major or minor themes in relation to the broad objective of understanding user experiences, while remaining open to discovery also [35].

### Ethical considerations

Ethical approval was specifically obtained for this study from the Ethics Committee of the Mahatma Gandhi Institute of Medical Sciences, Wardha, India (MGIMS/IEC/OBGY/99/2013) and MAMTA Ethics & Review Board (MERB/Dec 2013/001). This study was conducted within the provisions of research with human subjects [37]. Participants were counselled and informed about the purpose of the study in their local language. Informed consent was obtained and duly signed (or with thumb impression, if illiterate) by all participants involved in the study. No incentive was provided for participating in the study. Privacy and confidentiality were maintained throughout the study. All transcripts were held securely by the principal investigator and not returned to participants. All participants were provided with pre- and post-test counselling, and linked to follow-up care after confirmatory tests at the ICTC. All participants testing HIV positive were assessed for ART eligibility based on their CD4 counts, and were provided with ART and follow-up, as recommended by the World Health Organization (WHO).

## Results

### Characteristics of study participants

**Figure 1:**
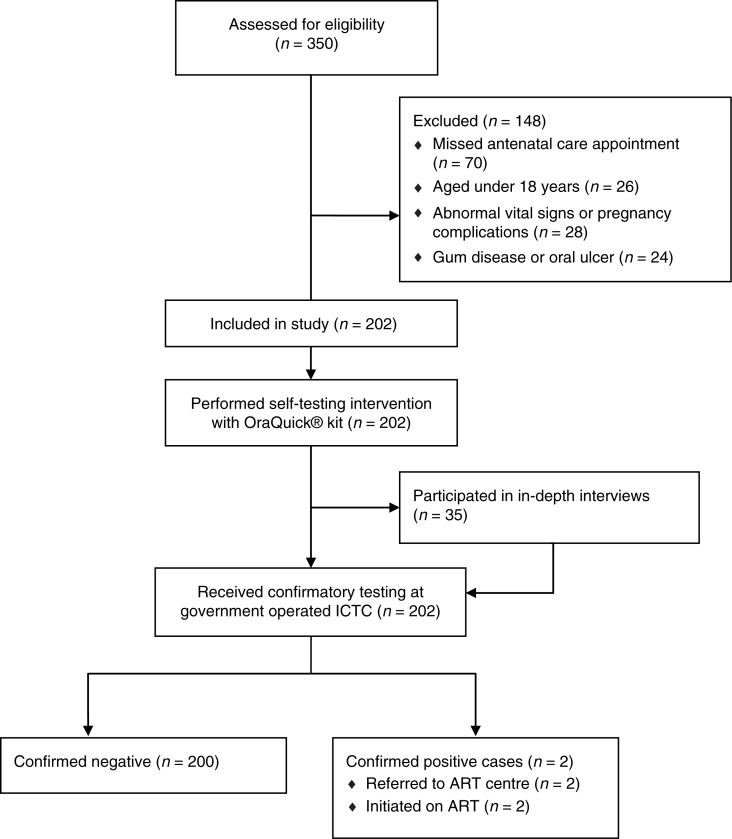
Recruitment and flow of participants in the study.

Of the 350 pregnant women registered in the facility during the study period, 202 met the inclusion criteria. Potential participants were excluded on the basis of an age of less than 18 (n=26), missing the antenatal clinic and therefore being absent during recruitment (n=70), having oral or gum disease, or active bleeding (n=24), or having abnormal vital signs (such as fever >38.5°C), or any other pregnancy complications that might have hindered informed consent (n=28) (Figure 1).

The median age of the 202 participants was 23 years, most were of low socio-economic status, 1% had no formal education, and 91.6% were not in formal employment. Although 95.5% reported that they had heard about HIV testing, only 28.2% had ever been tested for HIV before their current pregnancy (Table 1). Overall, 99.5% of women were nulliparous at the time of the study, although nearly 10% had a history of miscarriage or abortion. The characteristics of the sub-sample of 35 women who agreed to take part in the in-depth interviews are given in Table 1.

**Table 1:** Socio-demographic characteristics of study participants

	All participants (n=202)	Participants in qualitative interviews (n=35)
		
Characteristics	n (%)	n (%)
Age (years)		
Median age	23	23
Interquartile range	21–25	21–24
Social group^a^		
General	6 (3.0)	3 (8.6)
Scheduled caste	23 (11.4)	3 (8.6)
Scheduled tribe	6 (3.0)	2 (5.7)
Other caste groups	167 (82.7)	27 (77.1)
Education		
No formal education	2 (1.0)	1 (2.9)
Primary and middle education	18 (8.9)	4 (11.4)
Secondary education	129 (63.9)	21 (60.0)
Graduate or above	53 (26.2)	9 (25.7)
Husband's education		
No formal education	1 (0.5)	0 (0.0)
Primary and middle education	15 (7.4)	3 (8.6)
Secondary education	128 (63.4)	21 (60.0)
Graduate or above	58 (28.7)	11 (31.4)
Occupation		
Working/employed	17 (8.4)	3 (8.6)
Non-formally employed	185 (91.6)	32 (91.4)
Ever heard of HIV testing before?		
Yes	193 (95.5)	32 (91.4)
No	9 (4.5)	3 (8.6)
Ever tested for HIV before this pregnancy?		
Yes	57 (28.2)	9 (25.7)
No	145 (71.8)	26 (74.3)

a The study participants fall under different social groups as recognized by Constitution of India, namely scheduled castes, scheduled tribes, general and other caste groups.

### Acceptability

**Figure 2:**
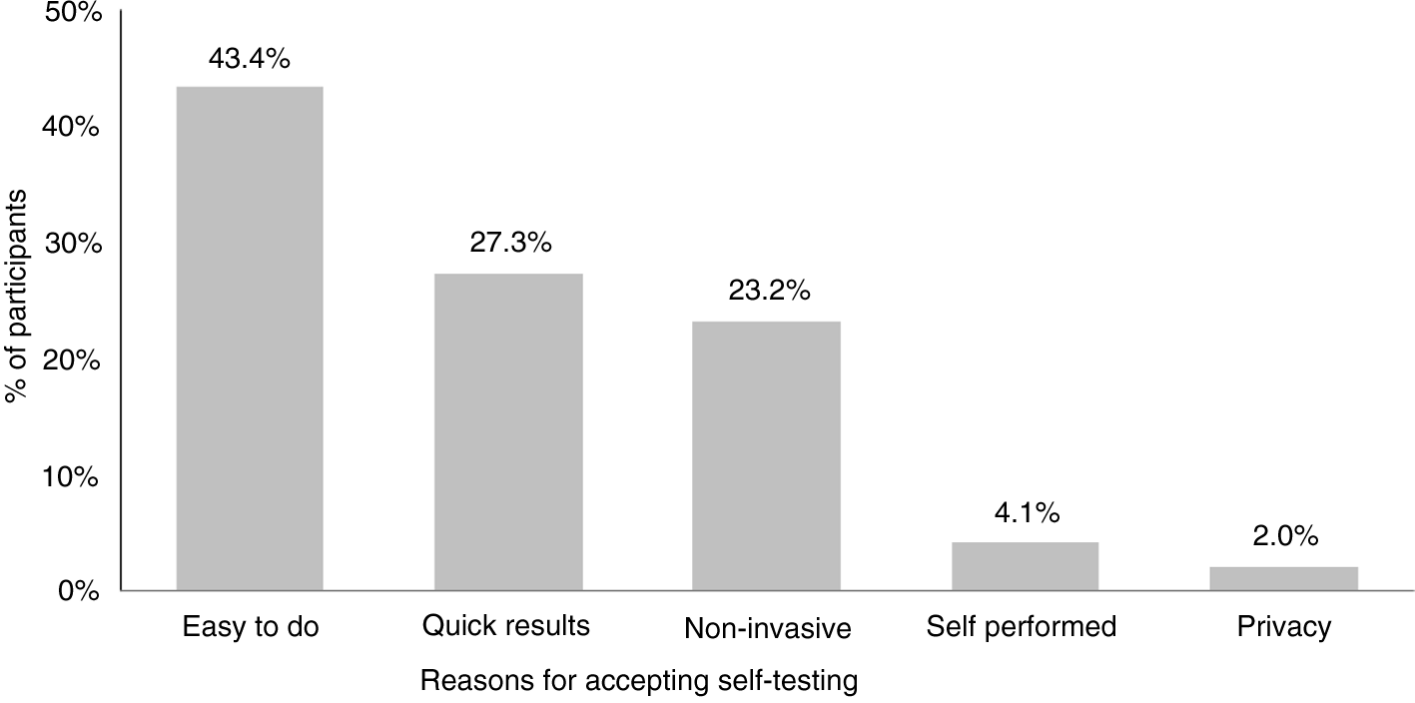
Reasons reported for liking the oral fluid-based HIV rapid self-test (n=198).

Acceptability of the oral fluid-based HIV test was high among pregnant women. Of the 202 offered the test, 100% accepted it as a screening tool while fully understanding that they would need to undergo confirmatory testing at the ICTC. When the pregnant women were asked whether they liked the test, 198 (98.0%) responded affirmatively. The most common reasons for this were that it was “easy to do” (43.4%), they got “quick results” (27.3%), and the test was “non-invasive” (23.2%). However, four women reported that they did not like the test because they felt it could not be as accurate as a blood-based test, or did not trust that a saliva test could be used for HIV screening (Figure 2).

The counselling process was perceived to be beneficial and necessary: 98% of pregnant women reported that they needed pre-test counselling, and 90.6% of pregnant women stated that they had benefited from post-test counselling. Overall, 194 (96.0%) of pregnant women tested reported that they would recommend this kit to other people, and 195 (96.5%) of those tested thought that the test kits should be sold in public outlets (Table 2).

**Table 2:** Acceptability and perceptions of oral fluid-based HIV rapid testing and counselling options among pregnant women

Variables	Affirmative responses (%) (n=202)
Pre-test opinions	
Pre-test counselling is required	198 (98.0)
Post-test opinions	
Liked the test kit	198 (98.0)
Tests kits should be sold in public outlets	195 (96.5)
Would recommend this kit to other people	194 (96.0)
Benefited from post-test counselling	183 (90.6)

Qualitative data provided additional insights into the factors that influenced acceptability of oral fluid-based HIV testing. Thematic analysis of the qualitative data revealed a range of facilitating factors and barriers to using oral testing (Table 3).

**Table 3:** Emerging themes on oral fluid-based HIV testing and implications for programming and research

Issue/coding concept	Major themes	Minor themes	Implications for programmes and research
Understanding self-testing the procedure	Clarity of test instructions	Literacy levels	Catering for illiterate populations may require adjusting instructions (e.g. using pictorials)
Acceptance and performance of the test	Time-efficiency Non-invasiveness	Availability of self-test kits outside of the hospital	Some participants could not wait for the required 20 minutes to read the results. Research is needed to understand how this could affect large programmes
	Convenience	Painless	
Interpreting the result	Clarity of instructions	Visual aids	Interventions focusing on invalid and other incorrect results without compromising confidentiality are needed
Barriers to and fear of self-testing	Fear of incorrect results		Emphasis that oral testing is a screening test that requires confirmation is critical for increasing uptake

When participants were asked to provide reasons for liking or disliking the test, their responses emphasized ease of performance and the ability to get results quickly:

I liked the test because I got the result quickly. (Interview, 21-year-old pregnant woman, village K)

I liked the test because it was very easy to do and we got the report in half an hour. (Interview, 23-year-old pregnant woman, village D)

I liked the test as it took little time for the testing process and it gives a result very quickly. (Interview, 23-year-old pregnant woman, village N)

I liked the test and it is very good as I could know whether I am HIV positive. I like the new test because this test gives very fast result and it was very easy to do. (Interview, 20-year-old pregnant woman, village K)

Others emphasized the fact that they did not have to provide a blood sample for the test:

I didn't have to give blood for testing, which helped me in reducing the fear and trouble. (Interview, 23-year-old pregnant woman, village P)

There was no need to give blood sample for this test, hence it was easy to use. (Interview, 25-year-old pregnant woman, village D)

### Sensitivity and specificity

Both sensitivity and specificity were found to be 100% for 201 tests. According to health workers’ interpretation of the oral test results, two were HIV positive and 199 were HIV negative. These results were then confirmed by an HIV test conducted at the ICTC. One oral test was deemed invalid by the supervisor and was excluded. The CD4 counts of the two HIV-positive participants were 245 and 48 cells/mm^3^, respectively, and both were initiated on a tenofovir, lamivudine and efavirenz combination ART regimen.

### Concordance

Of the 202 tests, 199 (98.5%) had concordance with a Cohen's Kappa (κ) value for inter-rater agreement of κ=0.566 with *p<*0.001 (Table 4).

**Table 4:** Inter-rater agreement between users (pregnant women) and supervisors (healthcare worker) on interpretation of oral fluid-based HIV test results

		Supervisor result			
			
κ=0.566, *p*<0.001		Positive	Negative	Invalid	Total
					
User result	Positive	2	0	0	2
	Negative	0	197	1	198
	Invalid	0	2	0	2
Total		2	199	1	202

### Feasibility

**Figure 3:**
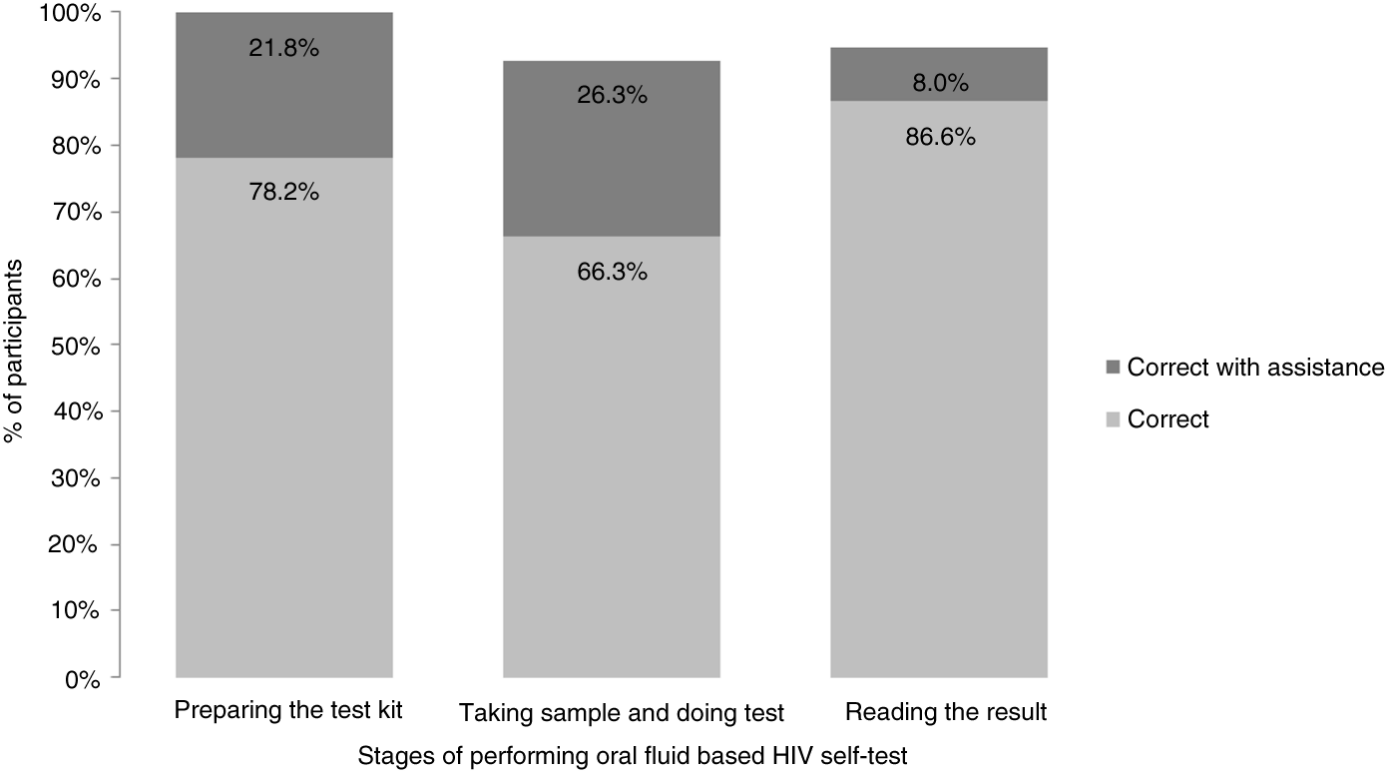
Proportions of participants who performed the test correctly.

In the study, documented errors were considered in each of the three main steps: (1) preparing the test kit, (2) taking the sample and doing the test and (3) reading and interpreting the result. An average 18.7% of participants required the assistance of a supervisor. Observations by the auxiliary nurse midwives showed that three participants swabbed their upper and lower gums incorrectly, and 15% required repeat instructions or another form of assistance to swab their gums correctly. With assistance, all of the test kits were prepared correctly, 92.6% of samples were taken correctly, and 94.6% of the tests results were read correctly (Figure 3).

Overall, 95.5% of participants reported being “confident that they performed the test correctly,” and 92.6% agreed that the “test kit instructions were easy to understand.” A small proportion of study participants (7.4%) reported some difficulty in understanding the test kit instructions. Three of the 202 participants waited for less than 20 minutes to read the oral test results, despite being told by health workers to wait for 20 minutes. Most participants suggested that the test was easy to perform, emphasizing the importance of the verbal instructions that were given before conducting the test. One participant remarked that she “did not experience any difficulty or trouble during test performance and did not commit any mistake, [because she] listened to the instructions very carefully given during pre-counselling” (Interview, 23-year-old pregnant woman, village K). Another participant echoed these remarks, stating that she “properly followed all the instructions given during pre-counselling,” and that in her view, “this test was not difficult for any woman to perform” (Interview, 23-year-old pregnant woman, village P).

When the participants were asked about the most difficult step to perform, “taking the sample and inserting the sample in the buffer solution” was deemed to be the most difficult, as stated by 27 (13.3%) participants. One user remarked:

This test was very simple to do and I predicted the correct result. I did all the steps but required assistance while taking the sample. (Interview, 24-year-old pregnant woman, village K)

At the same time, there were also concerns among participants about making mistakes:

I will be able to know the result quickly, but I have a fear of getting wrong result if I happen to make any mistake while performing the test. (Interview, 21-year-old pregnant woman, village P)

Overall, qualitative data suggested that participants believed the test to be accurate, particularly because there was confirmation of the results from either a government testing centre or an auxiliary nurse midwife:

I think the test was accurate since the nurse also confirmed it. (Interview, 23-year-old pregnant woman, village D)

This test is accurate as the result that I got was same as result done by government centre [ICTC]. (Interview, 20-year-old pregnant woman, village K)

## Discussion

Our study demonstrates the successful use of supervised HIV self-testing among a sample of pregnant women attending antenatal services in a rural Indian hospital. We found supervised self-testing using a rapid oral fluid-based HIV test to be acceptable and feasible in this population, and high concordance of result interpretation between participants and specifically trained community health workers. Our study, conducted in a low-prevalence setting and with a small number of subjects, found 100% sensitivity and 100% specificity of OraQuick^®^ HIV kits, based on health worker and ICTC results. High levels of sensitivity and specificity have been reported in other individual studies [12–16] and pooled results [17].

Although other studies have explored the provision of rapid testing among pregnant women, and demonstrated high acceptance levels using oral fluid [23,38] as well as blood-based methods [38,39], they have all used provider-initiated approaches. Currently, the National AIDS Control Organisation (NACO) in India recommends using blood-based rapid HIV test kits in all ICTCs across the country [40]. The recommendation, however, does not extend to rapid oral fluid-based HIV tests, either at ICTCs or for self-testing. To our knowledge, this is the first study of supervised oral self-testing among pregnant women that demonstrates the potential use of supervised rapid oral fluid-based testing among this population. Moreover, our study has utilized a cadre of community health workers to perform the supervision of self-testing in India.

In our study, all 202 women who were approached for inclusion agreed to perform the self-test. Factors contributing to the test's high acceptability include the ease of conducting the test, the short time to results and the non-invasive sample collection. These factors are similar to those reported as promoting acceptance in other studies, including convenience, ease of use, time-efficiency and the procedure being painless [41–43]. A cultural preference for giving an oral fluid rather than a blood sample was identified in a previous study in India, although the “novelty” of the oral fluid-based OraQuick^®^ test was reported as a possible reason for the preference [18]. Unlike other studies [15,33,41] participants in our study did not report privacy as a significant motivator for self-testing, which may be related to a desire for social support that outweighs privacy as demonstrated in other self-testing studies [43].

Among interviewed participants, 2% stated that they did not like the test. Qualitative data suggested that this was most likely due to uncertainty over the test results rather than any characteristic of the test itself. Several studies have previously noted a lack of trust in the accuracy of self-test results due to the fear of possible user error [41,44,45], although one study in the United States found that users were more confident in self-test results from a rapid oral fluid-based test compared to results from a finger-prick test [19].

More than 90% of pregnant women in our study performed the self-test without error, resulting in high feasibility for self-testing overall, although just under one-fifth required some assistance. Most women reported that the “test kit instructions were easy to understand,” with 7.4% reporting some difficulty in understanding the test kit instructions. Taking the sample and reading the result were the stages where errors were most commonly documented in the study. Previous studies evaluating supervised self-testing using oral fluid-based HIV tests, including in resource-poor settings, have documented a similar range of errors in conducting the tests [14]. In a study conducted in Malawi, Choko *et al*. [32] documented errors in sample collection and treatment, and interpretation of result, and identified the need for supportive supervision. Another study from the United States reported between 5 and 10% of users had difficulties in sample collection, reading test instructions and result interpretation [19].

In our study, a high concordance rate of 98.5% in result interpretation between participants and auxiliary nurse midwives was observed. We attribute this strong concordance to the test instructions and pictorial illustrations provided to each participant before they performed the self-test. While no false negative or positive results were reported, one result was deemed to be invalid by a trained health worker, most probably due to a defective kit or incorrect procedure. In addition, two test results were interpreted as invalid by participants when they were, in fact, negative. This is consistent with observations from a recent study in Singapore in which incorrect interpretation of results as invalid was the most common error in reading test results [33]. These findings underscore the observation that despite the general high accuracy of oral-based rapid tests, there is still the chance of a false negative, false positive, or non-reactive result [46].

Given the potential negative consequences of an incorrect result [47], strategies are required to mitigate the incidence and impact of incorrect results. As this was a feasibility study, all the women in our study were aware that HIV self-testing was a screening tool and that a follow-up test would be required to confirm results. In our study, confirmation was performed against an agreed reference standard, as recommended [46]. However, the role of confirmatory testing outside of research studies should be examined to inform policy and programs. A recent review also highlighted a need for retesting in situations of faintly positive lines, which can occur during the window period [14]. This is particularly important, given the reported small but significant false negative results from studies reporting high specificity: for example, in Singapore [33] and Malawi, where prevalence of false-negatives was 6 in 1000 within a recent large-scale community-based self-testing programme [13]. Although it did not affect the acceptance rates in our study, our observation that some participants found it difficult to wait for the required 20 minutes before reading the results suggests the need for careful supervision, especially in situations where participants are pressed for time or are required to take two tests, as was the case in our study. Further research may be needed to understand how this could affect error rates in large programmes outside of study settings. More broadly, as Wong *et al*. emphasize [48], quality assurance and regulation of test kits themselves will be critical in minimizing erroneous results.

In view of on-going debates comparing supervised versus unsupervised self-testing, our study employed a supervised self-testing approach that ensured practical, on-the-spot support immediately following self-testing, with identified ethical advantages over an unsupervised approach [49], and overcame potential barriers related to literacy for a minority of participants. Supervision enabled all participants to be linked directly to both pre- and post-test counselling, as well as to immediate referral for confirmation. Two pregnant women confirmed to be HIV positive were immediately linked to the nearest ART centre for treatment and care. The counselling linkage responded to the felt needs of the pregnant women themselves, as 98% of them reported the need for pre-test counselling and 90% felt that post-test counselling was beneficial. Similarly, in other studies participants have welcomed the integration of pre- and post-counselling into the testing process [32,33]. These observations suggest that a supervised approach can overcome some of the disadvantages of non-supervised self-testing related to potential lack of counselling services, delayed linkage to care and barriers related to illiteracy [14,49–51].

Utilization of community health workers, such as auxiliary nurse midwives, rather than nurses or doctors, to provide supervision, as was the case in this study, may mitigate an often-cited disadvantage of supervised self-testing related to the need for scarce healthcare professionals to be available to observe the test [14,15]. In India, auxiliary nurse midwives are widely available in healthcare centres at the village level, and their utilization in supervising self-testing could ease the workload on doctors and nurses. This strategy could reduce the human resource cost for screening services, while achieving task shifting. While this potential exists, we are also cognisant that the provision of oral-based self-testing has financial implications [46,52]. Although recent evidence suggests that it is a cost-effective approach [53], it costs US $4 per test for this study, indicating a need to ensure that HIV tests remain affordable.

### Limitations and implications for future research

The generalization of our findings is limited by a large number of participants being excluded from the study, including those with oral ulcers, gum disease, abnormal vital signs (such as fever >38.5°C), other pregnancy complications, and those who missed their antenatal care appointments. In rural settings of Maharashtra where this study was conducted, nearly 30% of pregnant women do not complete the recommended four antenatal care visits [54], which is slightly higher than the 20% observed in our study. However, it was not feasible to track women who missed their antenatal care appointments for follow-up visits as the study was conducted at a tertiary-level hospital where pregnant women visit from far-off places. Because of the long distance to the hospital and other socio-economic factors preventing women from antenatal care, it is unknown whether the results among the excluded groups would have been similar to those who participated in the study. Similarly, the qualitative sub-sample was small and self-selected on the basis of participant availability and willingness to participate. Thus the motivations and perceptions of self-testing identified in this study may not be representative of all the women in the study or study area. These issues would need to be addressed in follow-up research. Similarly, future research could explore the use of oral self-testing for partner testing.

As routine HIV testing was already acceptable to most pregnant women attending the antenatal clinic at the hospital prior to our study, we could not assess the impact of the intervention on rates of uptake. It is also possible that the presence of community health workers and researchers may have influenced participants’ testing procedures or their questionnaire responses.

Nevertheless, the high levels of acceptance suggest that supervised self-testing does not deter HIV testing at the study site, and may provide an opportunity to extend it to other health facilities. We report findings from supervised self-testing and acknowledge that we have not compared this directly to unsupervised self-testing. This is an area where follow-up research would be useful.

Although our study explored the potential use of supervised rapid oral fluid-based testing among pregnant women in health facilities, the majority of women in the study reported that they would recommend the test kit to other people, and most suggested that test kits should be sold in public outlets. These data are similar to findings elsewhere [33] and suggest that the OraQuick^®^ kits could be useful beyond the hospital setting. In this regard, it is important that the healthcare workers are equipped to deal with those who test positive in the field and require further confirmation and linkage to care. Future research could explore these implementation issues.

Lastly, the small number of women enrolled in the study, combined with the low prevalence of HIV among this population, limits the extent to which definite conclusions may be drawn in relation to sensitivity and specificity. Because sensitivity and specificity are indicators of test performance, these measures were derived using the health workers interpretation as the index test result rather than the participants’ interpretation. Reported sensitivity and specificity measures may have been slightly lower if the participants’ interpretations were assumed to be the index tests.

## Conclusions

With less than 40% of pregnant women being tested for HIV in India, innovative strategies are required to ensure the successful rollout of India's commitment to the B+ option strategy, in which early identification and initiation of ART is recommended among HIV-positive pregnant women. The results of this study, which utilized a cadre of community health workers known as auxiliary nurse midwives, rather than formally trained staff nurses and doctors, demonstrate that facility-based, supervised HIV self-testing could be feasible for Indian and other contexts in which a lack of adequate trained human resources impedes access to HIV testing. It is especially important to target pregnant women for successful prevention of mother-to-child HIV transmission.

As yet, there is no normative guidance from WHO on self-testing, and policy development varies across countries [48]. Some high-prevalence countries have included HIV self-testing in their national policy [55], but other countries, including India, have not yet approved self-testing within their national programmes. Regulatory approvals for test kits may also be required. For implementation to go forward, policymakers need to weigh up the potential advantages, as well as the risks of self-testing within their specific context [48]. Our study aimed to support this discussion in the Indian context. This is particularly relevant given that our study points to a potential use for self-testing outside of health facilities in the future, a strategy that has been found to be acceptable in other contexts [32].

## Supplementary Material

Feasibility of supervised self-testing using an oral fluid-based HIV rapid testing method: a cross-sectional, mixed method study among pregnant women in rural India
