# Supplementary material for: Feasibility of supervised self-testing using an oral fluid-based HIV rapid testing method: a cross-sectional, mixed method study among pregnant women in rural India
Source: J Int AIDS Soc. 2016 Sep 12;19(1):20993. doi: 10.7448/IAS.19.1.20993 (PMC5023853; doi:10.7448/IAS.19.1.20993)
Supplement: Feasibility of supervised self-testing using an oral fluid-based HIV rapid testing method: a cross-sectional, mixed method study among pregnant women in rural India [file JIAS-19-20993-s001.pdf]

Additional File 1: Orientation Guide of Self Testing

1.1. To start with

1. Participants were informed that they must not take any chewing materials, drinks and eatables at least 15 -20 minutes before the self-testing procedure.
2. Participants need to wash their hands with soap before and after the test.
3. If any participant has cut in the hand / finger, they must use disposable gloves.

Note:

1. Healthcare worker must have a watch/ timer capable of timing 20 to 40 minutes
2. The place where test will be conducted must be clean. Damp, dusty environment should be strictly avoided. Eating/drinking should not be allowed in that place. The place must have profuse light source.
3. A biohazard waste container should be placed in the testing room

1.2. Knowing the test Kit

About the test kit the participants should be told followings

1. Inform the participants that the test should be performed in temperature ranged from 15°C- 37°C. Thus if stored in refrigerator, the kit should be brought to operating temperature. They should be advised to keep the kit outside the fridge for few minutes to let it get ambient temperature.
2. Let the participant be familiarized with materials inside the kits. The following picture will be used

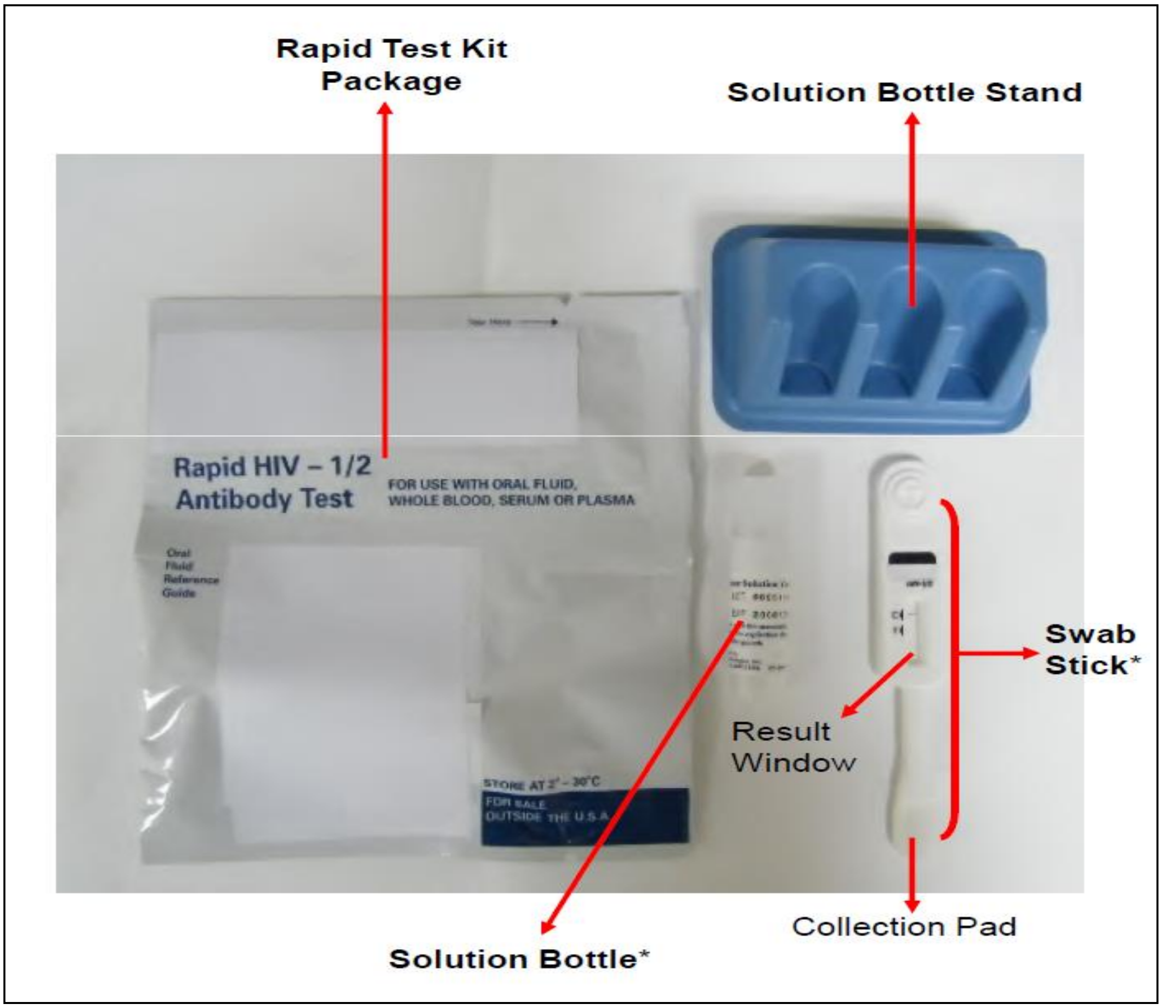

1.3. Using the Kit

The healthcare worker should describe the test procedure in following 10 simple steps showing the picture

1. Place the solution bottle stand on the table

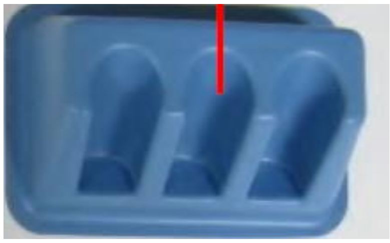

2. Open the package from the ridge from one side to other

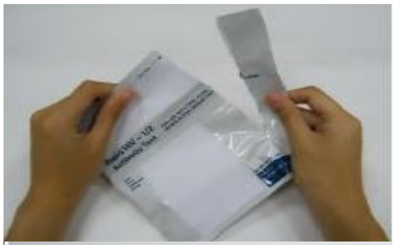

3. Remove solution bottle first from the package

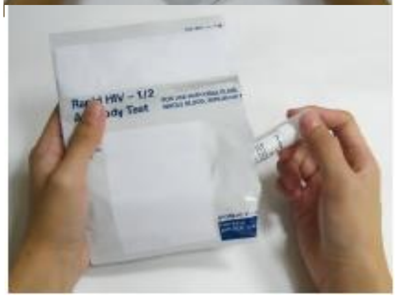

4. Remove the cap carefully and avoid spilling

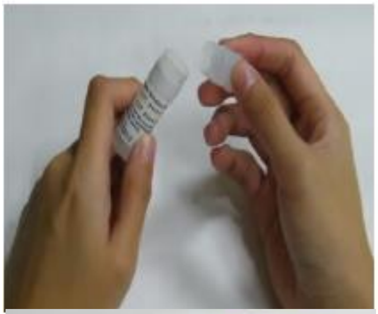

5. Place the bottle on the solution bottle stand

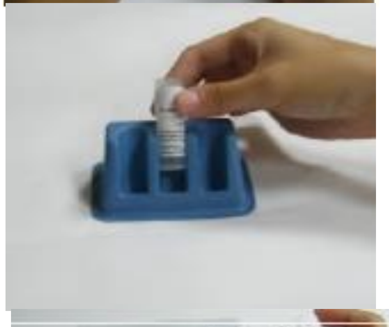

   - ***Do not force the bottle inside the stand***
   - ***Use the stand provided in the kits only. Do not use other stand***

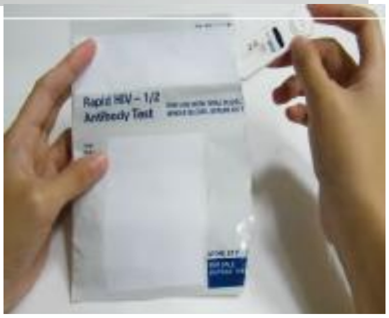

6. Remove the swab stick from the package

7. Hold the collection stick as shown in the picture. Do not touch the red circled part

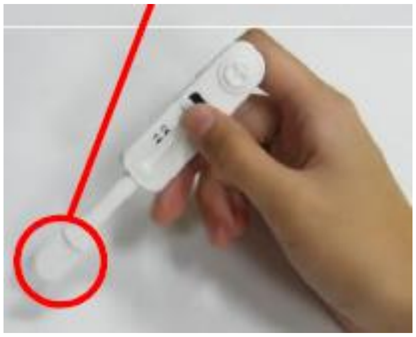

8. Swab around the upper gum with the collection pad. Avoid tongue, cheek and other parts of mouth

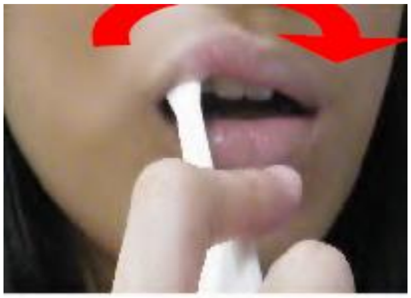

9. Swab around the lower gum with the collection pad. Avoid tongue, cheek and other parts of mouth

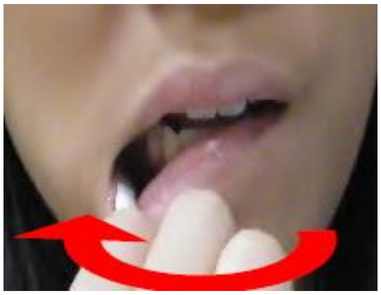

10. Place the stick in to the solution bottle

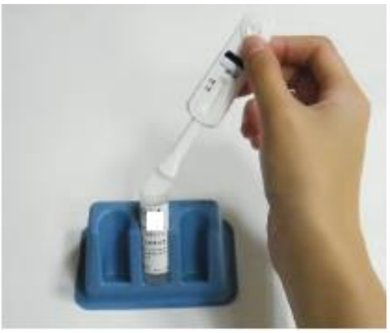

YOUR TEST PROCEDURE IS DONE

11. Inform the women to note the time when she dip the stick inside the bottle and keep the stick in following position for 20 minutes

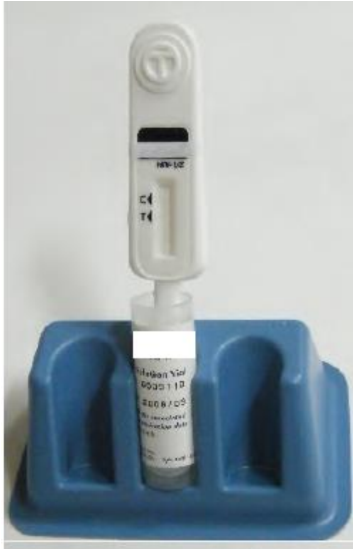

- ***Do not read the result before 20 minutes and after 40 minutes***

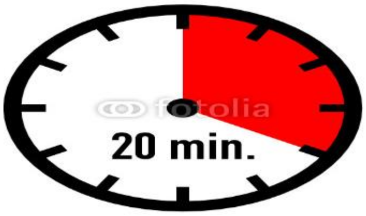

1.4. How to Read the result:

Show the following pictures to the participants and make them understand the meaning of each. Result should be noted after 20 minutes and before 40 minutes of the test.

1. **Negative**

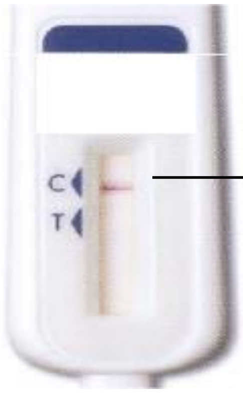

Only one line next to C

2. **Positive**

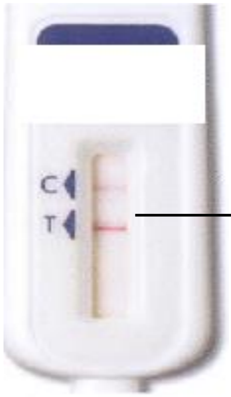

Two lines, one in c and another in T. No matter how faint one is

3. Invalid

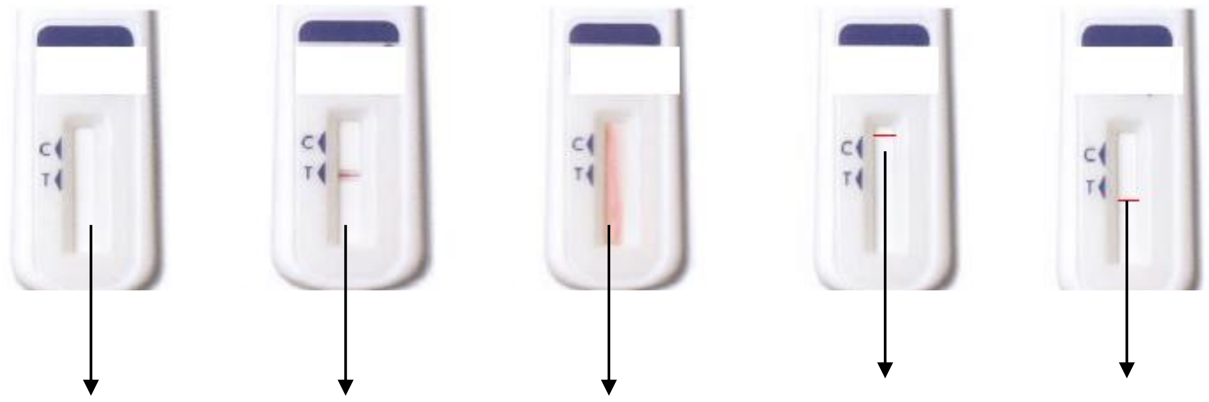

Note: The kit is not reusable. Orient the women that she should dispose all test components after use.
